# Pervasive Genotypic Mosaicism in Founder Mice Derived from Genome Editing through Pronuclear Injection

**DOI:** 10.1371/journal.pone.0129457

**Published:** 2015-06-08

**Authors:** Daniel Oliver, Shuiqiao Yuan, Hayden McSwiggin, Wei Yan

**Affiliations:** Department of Physiology and Cell Biology, University of Nevada School of Medicine, Reno, Nevada, United States of America; Osaka University, JAPAN

## Abstract

Genome editing technologies, especially the Cas9/CRISPR system, have revolutionized biomedical research over the past several years. Generation of novel alleles has been simplified to unprecedented levels, allowing for rapid expansion of available genetic tool kits for researchers. However, the issue of genotypic mosaicism has become evident, making stringent analyses of the penetrance of genome-edited alleles essential. Here, we report that founder mice, derived from pronuclear injection of ZFNs or a mix of guidance RNAs and Cas9 mRNAs, display consistent genotypic mosaicism for both deletion and insertion alleles. To identify founders with greater possibility of transmitting the mutant allele through the germline, we developed an effective germline genotyping method. The awareness of the inherent genotypic mosaicism issue with genome editing will allow for a more efficient implementation of the technologies, and the germline genotyping method will save valuable time and resources.

## Introduction

The recent advent of genome editing technologies has promised the ability to provide efficient and accurate genetic manipulations. These technologies rely on the basic mechanism of non-specific endonucleases that achieve target recognition through sequence-specific domains, which can be either proteins [Zinc Finger Nucleases (ZFNs) and Transcription Activator-Like Effector Nucleases (TALENs)] or RNAs [CRISPR-Associated Protein 9 (Cas9)/Clustered Regularly Interspaced Short Palindromic Repeats (CRISPR)] [1, 2]. Thus, these molecular amalgams can be engineered to target any known genomic sequence within their loose constraints, thereby inducing genetic manipulations through double stranded DNA breaks (DSBs) [3]. Once DSBs are generated, the cellular DNA repair machinery is recruited, defaulting to the error-prone, non-homologous end joining (NHEJ) repair mechanism; or alternatively, if a repair template is provided, homology directed repair (HDR)[4] is preferred and utilized [5]. These two mechanisms result in random mutagenesis at targeted loci through NHEJ, or cause engineered insertions *via* HDR.

The three main genome-editing tools include zinc finger nucleases (ZFN), transcription activator like effector nucleases (TALENs), and the clustered regularly interspersed palindromic repeats (CRISPR)/Cas9 (CRISPR associated) system [6]. ZFNs and TALENs both rely on their programmable, modular DNA-binding protein domains to acquire sequence-specific recognition. Alternatively, the CRISPR/Cas9 machinery recognizes sequence specificity through a 20bp chimeric single guide RNA (sgRNA), which binds DNA directly through complementary base pairing. The chimeric sgRNA contains the CRISPR RNA (crRNA) and transacting RNA (tracRNA): The crRNA contains the engineered sequence-specific DNA recognition sequence, while the tracRNA is essential for forming a complex with the CAS9 nuclease [7]. This complex can then initiate a double-stranded break (DSB) at the exact base pair conferred by the recognition of the sgRNA complex [8]. The modular specificity of Cas9/CRISPR has allowed it to stand out over TALENs and ZFNs, as the simple cloning steps to generate novel sequence-specific editing offer greater time- and cost-savings [9]. Thus far, Cas9/CRISPR has been demonstrated to work efficiently in a variety of organism, including plants, *Drosophila*, *C*. *elegans*, *Zenopus tropicalis*, zebrafish, mice, cows, and humans [10–15].

In mice, Cas9/CRISPR has been used to generate bi-allelic mutants in a single step through injecting the mix of gRNAs and Cas9 mRNAs into the pronuclei of fertilized eggs [16]. This approach has also been used to generate gene knock-ins and conditional alleles through HDR [17]. In addition, ZFNs have also been used in a similar capacity, both for “one-step” mutagenesis in the mouse zygote, and for HDR-mediated engineering [4]. Overall, these techniques are being quickly adopted for introducing engineered alleles into cells and whole animals.

While numerous reports in the past several years have utilized these technologies to generate deletion and insertion alleles, one unfortunate byproduct of their applications, genotypic mosaicism, has becoming increasingly evident [18]. A recent report [19], in which Cas9/CRIPSR was used to target *Tyr* gene has demonstrated that mice derived from pronuclear injections of gRNAs against this gene showed variable coat color, indicative of mosaicism. Here, we report that mosaicism appears to be a constant feature associated with generation of founder animals through pronuclear injection, as we have observed abundant mosaicism in ZFN-, Cas9 WT-, and Cas9 nickase-derived mutants, both through donor-independent NHEJ repair and HDR-directed insertions. Since the ultimate goal of these genetic manipulations is to establish stable breeding lines that faithfully transmit the mutant alleles, it is imperative to define the criteria for reporting “successful targeting”, as the numbers are highly variable between founder mice derived from pronuclear injections that possess detectable indels/engineered alleles in the tail DNA, and those that can truly transmit these alleles through the germline. Much like the traditional approach of homologous recombination, germline transmission is unequivocally essential. Since waiting for genotyping results for the second generation to confirm transmission can cost precious time, we report, here, an easy and convenient approach, i.e., germline genotyping, to expedite the identification of transmittable genome-edited alleles. Furthermore, our dissection of these mechanisms has led to our realization of a cruder, less labor-intensive means of identifying true transmittable, genome-edited alleles based on engineered band intensity and subclonal percentages. This knowledge is of help to researchers aiming to generate novel alleles through genome editing technologies, and save time and money in downstream characterization.

## Materials and Methods

### Ethics Statements

All animal experiments were approved by the Institutional Animal Care and Use Committee of the University of Nevada, Reno. All mice were housed and maintained under specific pathogen-free conditions with a temperature- and humidity-controlled animal facility in the Department of Lab Animal Medicine, University of Nevada, Reno.

### Generation of Cas9 bi-cistronic expression vectors

For *loxp* insertion, we chose to utilize the Cas9-nickase (D10A) mutant, as it can efficiently induce HDR-directed insertions, while mitigating off-target effects due to the DSBs that occur when using wild-type Cas9 [20, 21]. We used the Precision-X CRISPR/Cas9 Smart Nuclease System (SBI), with Cas9 nickase transcription under the CAG promoter, and sgRNA transcription under the H1 promoter.

Genes of interest were assessed for sgRNA specificity and efficiency using MIT’s CRISPR Design Tool (http://www.genome-engineering.org/crispr) (S1 Table). The pX330-U6-Chimeric_BB-CBh-hSpCas9 plasmid (Addgene) was used to drive the expression of WT Cas9 under the CBh promoter and the CAG enhancer, and the expression of sgRNA was driven by the strong early U6 promoter.

The vector (1μg) was digested with Bbs1 for 1 hour at 37°C. Meanwhile, sense and anti-sense oligos specific for each sgRNA were mixed at 10μM, heated to 95°C for 5 minutes, and allowed to cool to RT on the bench. Each “forward” oligo had a “CACCG” sequence added to the 5’ end, whereas each “reverse” oligo had an “AAAC” added to its 5’ end and an additional “C” added to its 3’ end (S1 Table). The adapters served to accommodate ligation into the BbsI-linearized vector, and to bolster T7 *in vitro* transcription (IVT). T4 DNA ligase and 10X buffer (NEB) were added to the digestion/ligation reactions, and these were incubated at 37°C for 1 hour. An aliquot of of each reaction (2μl) was then transformed into a tube of 5-alpha competent cells from NEB. Clones were selected, amplified, and their DNA was isolated using the Zymo MiniPrep Kit. An aliquot of each (2μl) was digested with Age1 and Bbs1 to screen for successful insertion of the sgRNA annealed oligos. Clones containing inserts were sequenced with the U6 forward primer: 5’-GAGGGCCTATTTCCCATGATTCC-3’.

### *In vitro* transcription of gRNAs

T7 promoter sequences were PCR-amplified onto the sgRNA forward sequences as such: 5’- GCGTTAATACGACTCACTATAGGGNNNNNNNNNNNNNNNNNNNN-3’ (N = 20bp gRNA forward sequence) (S1 Table). The additional G’s on the 3’ end of the T7 adapter were utilized to enhance the transcription efficiency of the T7 promoter. The pX330 universal gRNA reverse primer was used: 5’-AAAAGCACCGACTCGGTGCC -3’. Phusion Taq from NEB was used as follows: [98°C, 20sec; 72°C, 20sec] x35; 72°C, 1min; 4°C for holding. The resulting PCR products were run on 2.5% agarose TAE gels, stained with EtBr, and the bands were extracted at roughly 140bp. The DNA was recovered using Qiagen’s gel extraction kit. IVT reactions were performed following instructions in the NEB High-yield T7 *in vitro* Transcription Kit, but with an overnight incubation at 37°C. PolyA tails were added to the RNA products using NEB’s EPAP enzyme, following the directions detailed, with a 30-minute 37°C incubation period. After this reaction was completed, the RNA was recovered using Ambion’s RNA Easy kit, as per the instructions. Cas9 mRNA was ordered from Trilink (http://www.trilinkbiotech.com).

### Pronuclear injection and embryo transfer

FVB/NJ female mice at 4–6 weeks of age were superovulated by intraperitoneal injection of 5IU pregnant mares serum gonadotropin (PMCG) followed by 5IU human chorionic gonadotropin (hCG) at an interval of 48h, and mated overnight with C57BL/6J stud male mice of ~10 week old. Zygotes were collected after 20h of human chorionic gonadotropin (hCG) injection by flushing the oviducts followed by removing the cumulus cells. Zygotes with two pronuclei were washed three times using the M2 medium. After washing, the zygotes were transferred into KSOM+AA (Millipore, Cat# MR-121-D) medium in an incubator with air containing 5% CO_2_ at 37°C until microinjection. Microinjection was performed using an inverted microscope equipped with a microinjector (Eppendorf) following our standard protocol [22]. ~2~4pl of RNA solution containing 20ng/μl CAS9 mRNA, 10ng/μl gene-specific gRNA and 2ng/μl donor DNA (for knock-in strategy) was injected into the pronuclei and cytoplasm of each zygote using a microinjector (FemtoJet, Eppendorf). After injection, all zygotes were cultured for 1h in KSOM+AA medium at 37°C before being transferred into the oviductal ampullae (10~15 zygotes per oviduct) of 7–10 week-old female CD1 mice mated with vasectomized CD1 males at the previous night. The injection mix contained the following: 100ng/μl of each gRNA and 200ng/μl of Cas9 mRNA. For those injections containing DNA, 20ng/μl of HDR DNA was added to this injection mix.

### PCR-based genotyping

After birth, pups were genotyped using tail DAN and PCR. Tail lysates were prepared as described previously [23]. GoTaq 2X PCR master mix (Promega) was used with the genotyping primer pairs listed in S1 Table. All PCR conditions were as follows: 95°C, 2min; [95°C, 30sec; 58°C, 30sec; 72°C, 45sec] x 32; 72°C, 5min; 4°C forever. PCR products were run on 2% Agarose gels in 1X TAE buffer. After genotyping, the mutations identified through tail PCR were sub-cloned and sequenced to further confirm the mutation.

## Results

### Founders derived from zygotes subjected to Cas9/CRISPR-mediated loxp insertions display mosaicism

**Fig 1:**
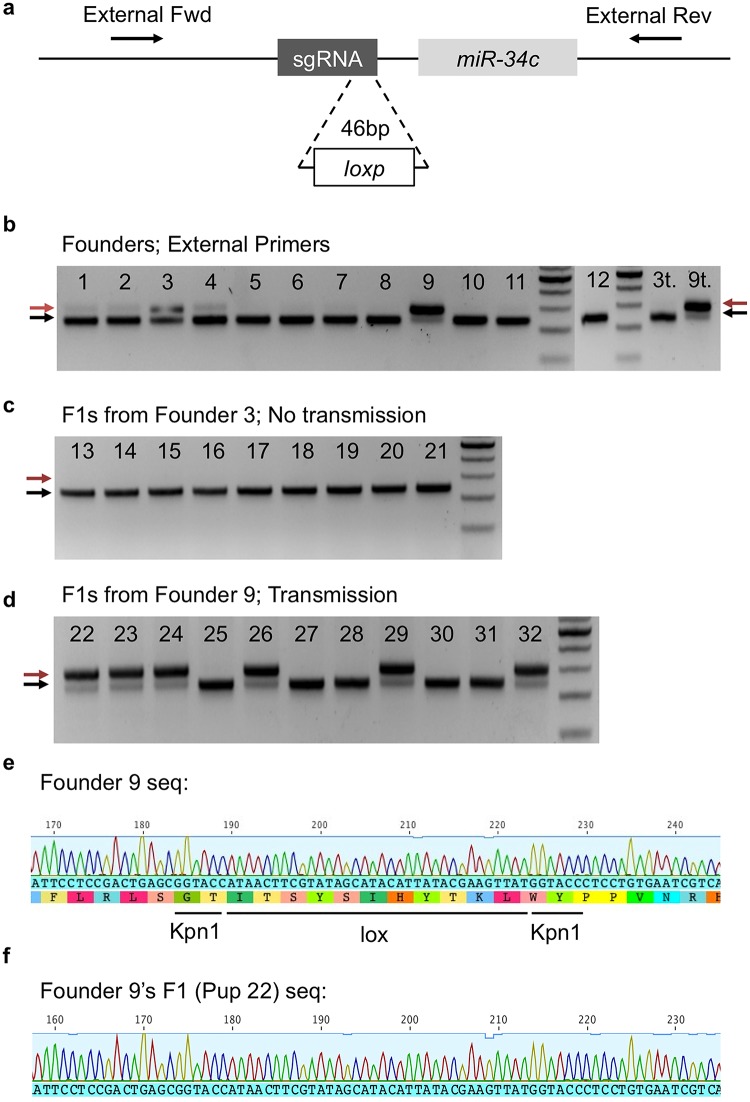
Cas9/CRISPR-mediated *loxp* sequence insertion adjacent to *miR-34c*. (a) Schematic representation of DNA sequence structure and genotyping primer design. Primers are indicated as orientation-specific arrows. The *loxp* insertion adds 46bp to the 288bp WT external amplicon size (334bp total, white box). *miR-34c* is indicated by the light grey box (77bp). The sgRNA target site is indicated by the dark grey box (20bp). The sgRNA was designed to target the wild-type sequence, with minimal effect on the HDR construct, or edited genomic sequence. (b) Agarose gel images showing amplicons using external forward (Fw) and revers (Rv) primers. WT bands are marked with black arrows, whereas the “*loxp* shift” bands are indicated using red arrows. Note that successful *loxp* insertion led to band shifts ranging from 288bp to 334bp. Germline genotyping assays were performed using the testes from Founder 3 (3t) and Founder 9 (9t) (t = testis). Founder 9 showed much greater abundance of the *loxp* shift bands than Founder 3. (c) Tail DNA PCR genotyping for F1 progeny of Founder 3, which was bred with WT females, showed no germline transmission of the *loxp* allele (Pups 13–21). (d) Tail DNA PCR genotyping results of F1 pups from Founder 9, which was crossed with WT females, showing successful germline transmission of the *loxp* allele. (e) Sequencing results of Founder 9 tail DNA, showing the successful *loxp* insertion, flanked by two engineered Kpn1 sites. (f) Sequencing results of tail DNA from Founder 9’s F1 progeny (Pup 22).

One of our primary goals was to generate loxp alleles in mice. To generate *miR-34b/c* flox mice, we first attempted to insert one *loxp* upstream of *miR-34c* (Fig 1a). Of the 12 pups obtained through our first round of pronuclear injections, five showed the PCR band corresponding to a heterozygous *loxp* insertion, whereas several displayed hardly any *loxp* band (Fig 1b). These amplicons were designed to span the region of the engineered *loxp* insertions, resulting in a band shift of 46bp in case of successful *loxp* insertions. Interestingly, we did not see an even distribution of band intensities (Fig 1b). It became clear that not all of the *loxp*/+ pups were behaving identically in terms of the abundance of the *loxp* allele. Namely, Pup 9 showed ~50% *loxp* allele colonies when whole PCR products were subcloned, while Pup 3 showed less than 5%, with the remainder being wild-type alleles. Furthermore, these clonal percentages correlated roughly with the relative band intensities of individual tail lysate genotypes (Fig 1b). Consistently, Pup 9 showed robust PCR genotyping detection of the *loxp* allele, whereas Pup 3 showed very faint bands by comparison (Fig 1b).

Curious about this phenomenon, we further bred these founders with WT to observe germline transmission of the *loxp* alleles. Interestingly, Pup 9 transmitted the *loxp* allele, while Pup 3 failed to display any germline transmission after numerous litters (Fig 1c and 1d), suggesting that Pup 3 contained much fewer, or probably no sperm, carrying the *loxp* allele. By comparing the initial tail lysate PCR genotyping results with the breeding data, it became clear that the mice that showed stronger *loxp* allele amplification were much more likely to demonstrate germline transmission (Fig 1b–1d). To further understand this phenomenon, we conducted germline genotyping experiments. For these, we used the same PCR genotyping protocol to genotype the DNA samples isolated from either the whole testis, or epididymal sperm. Consistent with our breeding data (Fig 1c and 1d), Pup 9 showed robust *loxp* allele amplification in the testis lysate samples (sample 9t in Fig 1b), whereas Pup 3 did not show any evidence of the *loxp* allele (sample 3t in Fig 1b). All of the *loxp* alleles were confirmed *via* sub-clonal sequencing (Fig 1e and 1f). The disparity in PCR genotyping results between tail and testis lysates demonstrates the genotypic mosaicism in Cas9/CRISPR-based genome editing.

### *Ubqlnl* homozygous deletion mice, identified through tail DNA genotyping, display germline mosaicism

**Fig 2:**
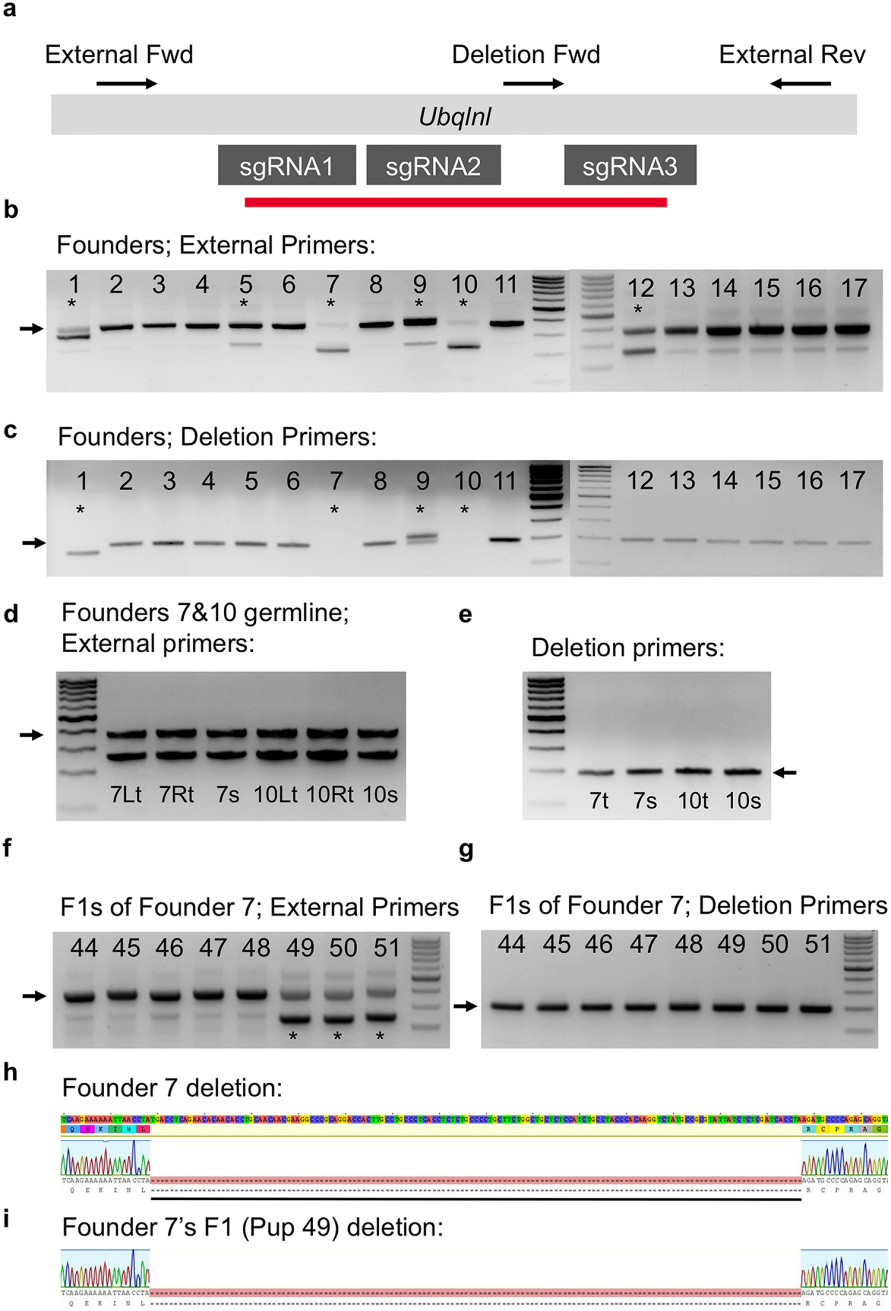
Cas9/CRISPR-mediated deletion in *Ubqlnl*. (a) Schematic representation of DNA sequence structure and genotyping primer design. Primers are indicated as orientation-specific arrows. The wild-type external amplicon is 376bp, while the deletion alleles vary in size, relative to the wild-type amplicon. The deletion forward (Fwd) primer, paired with the external reverse (Rev) primer, amplifies a 192bp amplicon in wild type. Deletion alleles that cover this region do not amplify. sgRNA binding sites are indicated by the dark grey boxes. The approximate regions of the deletions in Founders 7 and 10 are depicted by the red line. (b) The founder pups (1–17) were genotyped using the external primer set. The WT allele band is indicated by the black arrow. Any band, above or below, represents either an insertion, or deletion, respectively (*). (c) The founder generation was likewise genotyped with the deletion internal primer amplicon. (d) Founders 7 and 10 were germline genotyped, using the external primer set. (e) Founders 7 and 10 were germline genotyped, using deletion primer set (t = testis; s = sperm; L = left; R = right). (f-g) The F1 progeny of Founder 7 (deletion/deletion based on tail lysate genotyping) was likewise genotyped, and the results suggest mosaicism in Founder 7’s germline because some pups received the deletion allele from Founder 7, while others were homozygous wild type. (h) Representative sequencing results for Founder 7 using tail DNA. (i) Representative sequencing results for Founder 7’s F1 progeny using tail DNA. Sequences of the amplicons using external primers for Founder 7 and one of Founder 7’s progeny (F1 Pup 49) displayed a large deletion.

We encountered another case of false-positive genotyping results due to mosaicism, in which a founder mouse displayed a homozygous deletion of *Ubqlnl* based on tail DNA genotyping, but never transmitted the deletion allele to their offspring. We used a multi-gRNA approach and generated a ~150bp deletion in the only exon of *Ubqlnl* (Fig 2a). Of 17 founders, 6 had deletions that were detectable *via* PCR band shifts on agarose gels, using a primer pair flanking the gRNA target sites (Fig 2b). Interestingly, Founder 7 and Founder 10 did not display the larger wild-type bands (Fig 2b). We next used a PCR genotyping amplicon that would only detect the wild-type allele in this mouse, as the forward primer was located within the deletion, with the reverse lying just outside (Fig 2c). With this method, no wild-type allele was detected in the tail DNA of Founder 7 and Founder 10, whereas it was readily amplified in the other founder mice, even those with heterozygous deletions in this region (Fig 2c). Additionally, sequencing failed to detect any wild-type allele among 10 randomly chosen clones from Founder 7 and Founder 10, and all 20 clones analyzed were from the deletion alleles.

Given these data, we then bred Founder 7 and Founder 10 with WT females to expand these two lines, and also to test their fertility because the tail PCR genotyping results made us believe that we had obtained *Ubqlnl* homozygous mutant mice. Both of the so-called *Ubqlnl* homozygous deletion males demonstrated fertility, yielding normal litter sizes of 8 and 9 pups, respectively. To see whether the genotype is consistent between the tails and the germ cells, we further genotyped DNA isolated from the testis (t) and sperm (s) of Founder 7 and Founder 10 (Fig 2d and 2e). Interestingly, both founder mice showed evidence of both wild type and deletion alleles in both their testes and sperm, as indicated by the correct band sized on genotyping gels (Fig 2d and 2e). Therefore, these two founders displayed germline mosaicism despite that the tail genotyping suggested homozygous deletion. Consistent with this finding, tail DNA genotyping of F1 pups fathered by Founder 7 and Founder 10 revealed that many F1 pups were homozygous wild type, which would have been impossible if the two founders were truly homozygous (Fig 2f and 2g). We did note a lower, faint band in these homozygous wild-type pups, but this was subcloned and determined to be an amplification artifact. We sequenced the PCR products and the deletion alleles were confirmed in both founders and their offspring (Fig 2h and 2i).

To evaluate the possibility of genotyping disparity between the right and the left testis, we genotyping both testes from founders Founder 7 and Founder 10 (Fig 2d). As expected, both the right and left testes showed similar levels of wild type and deletion alleles. Identical genotyping results between the left and the right testes are expected because it is well known that both start to develop upon colonization by the same group of primordial germ cells (PGCs), which arise at ~E6.5 and reach the genital ridges at ~E9.5. Both time points are long after the initial couple of rounds of cell divisions (zygotes, 2-cell, 4-cell-8-cell, etc.), during which the Cas9/CRISPR machinery can possibly work. Thus, it is almost impossible for sperm in the left testis to be different from those in the right testis. Taken together, these data demonstrate that germline mosaicism can exist despite that tail DNA genotyping results conclusively show homozygous deletions. This finding emphasizes the need to directly genotype sperm or testis. Furthermore, it exhibits the necessity of waiting for germline transmission in F2 mice, rather than relying on tail genotyping results directly from founder mice. Lastly, the similar genotyping results between the right and the left testes is supportive of the idea that genotyping one testis is sufficient to give a reliable indication of the germline genotype.

### Founder mice derived from zygotes subjected to Cas9/CRISPR-mediated deletion of *miR-741* display mosaicism

**Fig 3:**
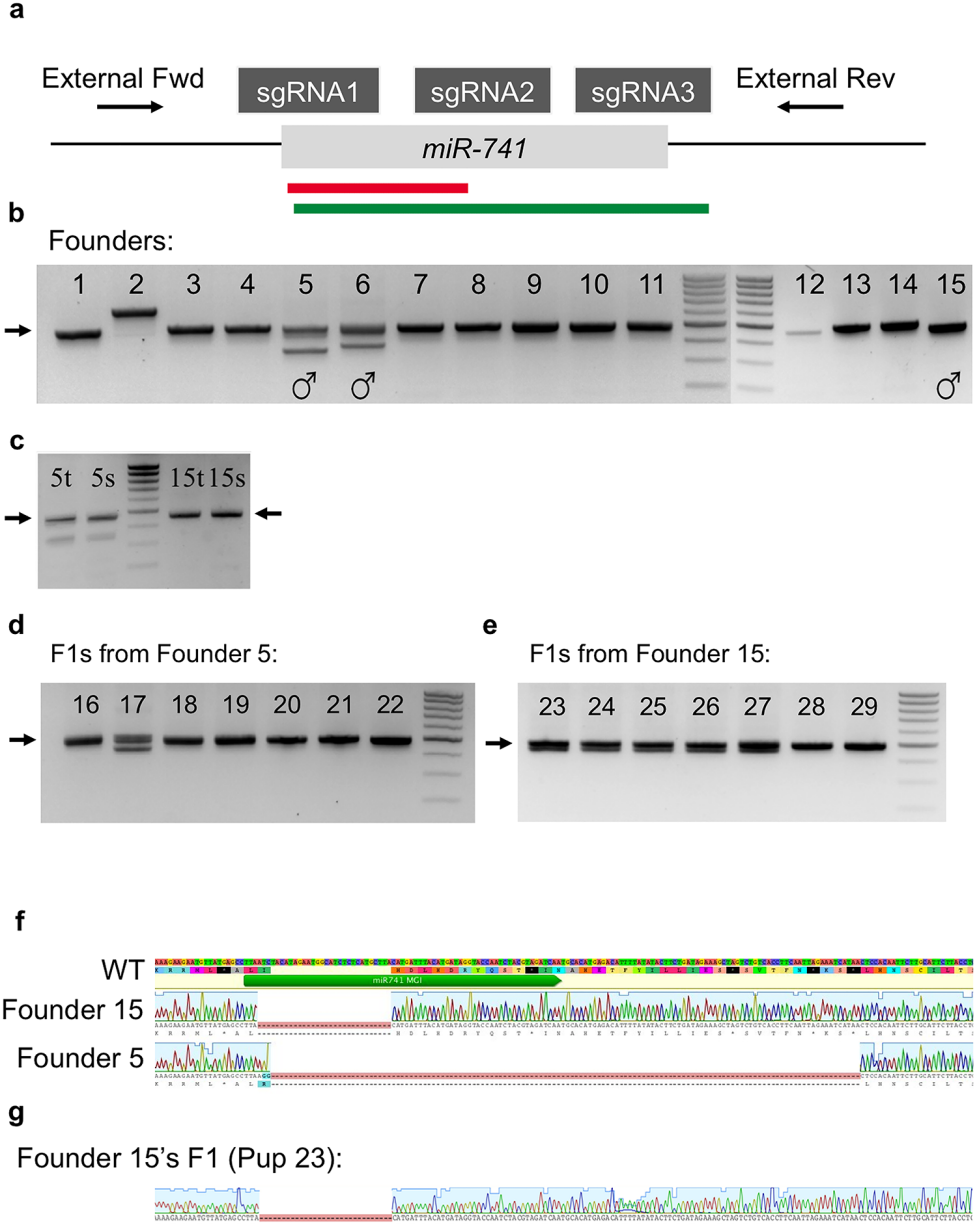
Cas9/CRISPR-mediated deletion of *miR-741* shows mosaicism in multiple forms. (a) Schematic representation of DNA sequence structure and genotyping primer design. Primers are indicated as orientation-specific arrows. The wild-type amplicon is 484bp, while the deletion alleles vary in size, depending on the NHEJ. sgRNA binding sites are indicated by dark grey boxes. *miR-741* is depicted as a light grey box. The approximate deletion in Founder 5 is depicted by the green line, whereas that of Founder 15 is depicted by the red line. (b) Agarose gel images showing tail DNA PCR genotyping for founder mice using the external primer set. The approximate band size of the WT allele amplicon is indicated by the black arrow. The deletion/insertion alleles were variable in size, depending on the founder mice; Founder 5 and 15 had a 132bp and a 30bp deletion, respectively. Founders 5 and 6 represent mosaic males, showing both wild-type allele amplification (upper band) and deletion allele amplification (lower band). (c) PCR germline genotyping results of Founders 5 and 15, showing the presence of the multiple alleles (t = testis, s = sperm). (d) PCR tail genotyping of F1 pups (F1 Pups 16–22) fathered by Founder 5. (e) PCR tail genotyping of F1 pups derived from Founder 15 (F1 Pups 23–29). (f) Representative sequencing results for wild-type miR741 (upper), Founder 5 (lower) and Founder 15 (middle). (g) Representative sequencing results for wild-type miR741 (upper) and F1 Pup 23 fathered by Founder 15.

The discovery of genotypic differences between tail lysate and germline transmission suggests prevalent mosaicism in founder mice derived from Cas9/CRISPR-based genome editing through pronuclear injection. We also discovered that mosaicism is prevalent within tail lysates themselves. For instance, we attempted to generate global deletion lines of *miR-741*, an X-linked miRNA. Again, we used a multi-sgRNA approach to induce deletions at this genomic locus (Fig 3a). Any male should present only one allele, since this is an X-linked gene. However, two males in our tail lysate PCR genotyping showed multiple alleles (Founders 5 & 6), representing deletion alleles and also wild-type alleles (Fig 3b). This is consistent with the idea that some cells in the tail were, in fact, genome-edited, while others contained intact wild-type alleles.

In order to further examine the utility of direct germline genotyping, we once again genotyped whole testis and sperm samples (Fig 3c). As expected, Founder 5 showed both wild-type and deletion alleles in both testis and sperm genotyping (Fig 3c). Thus, mosaicism at this locus was conserved in both the tail and the germline in this founder male. Alternatively, Founder 15, which showed no wild-type allele amplification in tail lysate genotyping, revealed no detectable wild-type allele in testis or sperm genotyping (Fig 3c). To date, this was our only example of a non-mosaic founder mouse, derived from Cas9/CRISPR genome editing. This demonstrates the possibility of obtaining a perfectly edited mouse through *in vivo* genome editing is rather low. In spite of germline confirmation, this mouse shouldn’t be used for characterization of the knockout phenotype because the potential for other mosaic tissues was still a possibility. By breeding Founders 5 and15 with WT mice, the mutant alleles were transmitted through the germline to the F1 progeny, which contained the mutant alleles in all cells and thus, were true *miR-741* mutant mice (Fig 3d and 3e). The PCR products from the genotyping assays were further sequenced, and the results confirmed the deletions (Fig 3f and 3g).

### Germline genotyping can predict the chance of germline transmission

One approach that we utilized, can potentially expedite confirmation of germline transmission of the engineered allele, at least for male founder mice. For this, we surgically removed one epididymis of the male, and used the sperm obtained therein for genotyping. Alternatively, one testis can be surgically removed, and genotyped likewise. For estimating the chance of germline transmission, sperm genotyping appeared to be more indicative than the whole testis genotyping, probably because sperm represent more pure male gametes with no or very little contamination of somatic cells. It is noteworthy that male mice with one testis surgically removed all displayed normal fertility (S2 Table). Additionally, this method provides usable tissue for preliminary characterization (e.g., immunohistochemistry, Western blot, qPCR, etc.) in case the phenotypic effect of the engineered allele is unknown beforehand. This method also allowed us to ascertain the likelihood of transmission in male founders derived from genome editing *via* pronuclear injections (Fig 1b–1d; Fig 2b–2g; Fig 3b–3d). In our experience, the detection of the engineered allele in sperm had a 100% correlation to the Mendelian segregation of the allele in the subsequent generation (i.e., ~50% of resulting pups contained the novel allele). After successful transmission to the offspring, all of the mutant alleles segregated following the Mendelian ratio in multi-generation breeding experiments. This germline genotyping method is mainly applicable to the males, as surgical removal of one testis is fairly trivial, yielding very high rates of survival and fertility (S2 Table). A similar approach could, in theory, be developed for female founders.

## Discussion

Genome editing technologies offer the promise of expedited generation of knockout and knock-in alleles, in all organisms tested so far. Since these technologies are still relatively new, best practices and methods for optimal downstream characterization are still largely needed. Here, we presented our experience with ZFN- and Cas9/CRISPR-mediated genome editing. Our data demonstrate the pervasive problem of mosaicism in founder mice when genome editing is carried out in zygotes through pronuclear injection. Although this finding does not impact the utility of the genome editing technologies, we do seek to bring attention to the frequent “false positive” founders, due to genotypic mosaicism present in both the tail and the germline. The awareness of these phenomena, and the methods we propose to detect them, will aid researchers using these techniques to a greater efficiency. Time and money spent tracking down false positives can be better invested in other exploits.

The realization of mosaicism resulting from the use of this technology is not surprising. Both Cas9/CRISPR and ZFNs rely on DNA and/or RNA delivery, commonly into the pronucleus of fertilized eggs. However, DSBs and the subsequent NHEJ-mediated deletions, or HDR-based insertions may not achieve full penetrance before the one-cell stage, and it has been shown that the injected DNA and RNAs persist and function beyond one-cell embryos [18, 19]. In this case, tissues and cells derived from those partially edited precursor cells would have different genotypes, thus yielding mosaicism not only in germline, but also in somatic tissues. Here, we demonstrated the impact of false positives in this context, but it could just as likely be the reverse scenario; pups identified as false negatives through tail lysate genotyping could actually be positive for the engineered allele in the germline. Thus, pups could be disregarded, when, in fact, they possess the sought-after engineered alleles in their germline. Given the widespread mosaicism in founder mice derived from pronucleus injection-based genome editing strategy, it is essential to treat the founders as chimeras, which should be subjected to further breeding to achieve germline transmission and to obtain true heterozygous and homozygous mutants for functional analyses.

Germline genotyping offers an efficient method for determining whether germline transmission will occur in founder mice. In lieu of such a technique, our best success was with estimating the likelihood of germline transmission based on the band intensity of PCR products for the *loxp* alleles in the tail lysates. Generally speaking, when we saw brighter “genome-edited” bands in tail DNA genotyping results, we usually observed higher percentages of germline transmission. This is especially useful for female founder mice, as germline genotyping is not as feasible as it is in their male counterparts. While this approach is not a guarantee, it at least increases the likelihood of selecting the founder mice with the highest chance of germline transmission, which means reducing animal care costs. Additionally, rather than waiting on false positives that don’t pan out, and having to do more injections months later, this method can be adopted to gain an estimate of the likelihood of germline transmission, and thus, inject more zygotes sooner, rather than later, if it is deemed necessary.

## Supporting Information

S1 Table DNA oligonucleotide sequences used for Cas9/CRISPR genome editing, *in vitro* transcription, and genotyping. (PDF)

S2 Table Breeding results for founder pups depicted in this manuscript. (PDF)
