# Supplementary material for: Pervasive Genotypic Mosaicism in Founder Mice Derived from Genome Editing through Pronuclear Injection
Source: PLoS One. 2015 Jun 8;10(6):e0129457. doi: 10.1371/journal.pone.0129457 (PMC4459985; doi:10.1371/journal.pone.0129457)
Supplement: S1 Table — (PDF) [file pone.0129457.s001.pdf]

Gene/sgRNA for pX330 Ligation:	Fwd sgRNA Oligo (5’-3’):
miR-34c-lox	caccg CGATTCACAGGAGGCTCAGT
UbqlnL gRNA1	caccg GTTGCAGGTGTTGTGTTCTG
UbqlnL gRNA2	caccg GAGAGGTGAGGGCAGGCAAG
UbqlnL gRNA3	caccg GGTGATCGAGAGATAATACG
miR-741 gRNA1	caccg GAGTTATGATTTCTAATTGA
miR-741 gRNA2	caccg ATGCCATTCTATGTAGATTA
miR-741 gRNA3	caccg GAGCCTTAATCTACATAGAA

IVT Oligo:	Sequence (5’-3’):
T7-*miR-34c-lox* gRNA Fwd	GCGTTAATACGACTCACTATAGGG CGATTCACAGGAGGCTCAGT
T7-*UbqlnL* gRNA1 Fwd	GCGTTAATACGACTCACTATAGGG GTTGCAGGTGTTGTGTTCTG
T7-*UbqlnL* gRNA2 Fwd	GCGTTAATACGACTCACTATAGGG GAGAGGTGAGGGCAGGCAAG
T7-*UbqlnL* gRNA3 Fwd	GCGTTAATACGACTCACTATAGGG GGTGATCGAGAGATAATACG
T7-*miR-741* gRNA1 Fwd	GCGTTAATACGACTCACTATAGGG GAGTTATGATTTCTAATTGA
T7-*miR-741* gRNA2 Fwd	GCGTTAATACGACTCACTATAGGG ATGCCATTCTATGTAGATTA
T7-*miR-741* gRNA3 Fwd	GCGTTAATACGACTCACTATAGGG GAGCCTTAATCTACATAGAA
pX330 Universal gRNA Rev	AAAAGCACCGACTCGGTGCC

Genotyping Gene:	Genotyping Fwd Oligo (5’-3’):
miR-34c-lox	ACCCCACACAAATTGATACATTGT
UbqlnL	AGGCCTCAGCTGAGCCACAT
UbqlnL Deletion Fwd	TTCTGGCTGCTCTCCATCTG
miR-741	CAATGATCTTCCAAGGCCAC

Rev sgRNA Oligo (5'-3'):
aaacACTGAGCCTCCTGTGAATCGc
aaacCAGAACACAACACCTGCAACc
aaacCTTGCCTGCCCTCACCTCTCc
aaacCGTATTATCTCTCGATCACCc
aaacTCAATTAGAAATCATAACTCc
aaacTAATCTACATAGAATGGCATc
aaacTTCTATGTAGATTAAGGCTCc

Genotyping Rev Oligo (5'-3'):
TTTTCTGAGTCTAGTTACTAGGC
CACTAAGGTGTCATCAGCTACTGT
GGGCAGATCTGAGAATAGGT
