# Supplementary material for: Pervasive Genotypic Mosaicism in Founder Mice Derived from Genome Editing through Pronuclear Injection
Source: PLoS One. 2015 Jun 8;10(6):e0129457. doi: 10.1371/journal.pone.0129457 (PMC4459985; doi:10.1371/journal.pone.0129457)
Supplement: S2 Table — (PDF) [file pone.0129457.s002.pdf]

**Founder Mouse**	**F1 litter size (M/F)**
miR-34c-lox FP3 (testis removed)	9 (5/4)
miR-34c-lox FP9 (testis removed)	11 (4/7)
UbqlnL FP7 (testis removed)	8 (5/3)
UbqlnL FP10 (testis removed)	10 (5/5)
miR-741 FP5 (testis removed)	7 (2/5)
miR-741 FP15 (testis removed)	7 (4/3)
